# Mechanism of floral scent production in *Osmanthus fragrans* and the production and regulation of its key floral constituents, β-ionone and linalool

**DOI:** 10.1038/s41438-019-0189-4

**Published:** 2019-09-07

**Authors:** Yuanji Han, Hongyun Wang, Xiaodan Wang, Ke Li, Meifang Dong, Yong Li, Qian Zhu, Fude Shang

**Affiliations:** School of Life Sciences, State Key Laboratory of Cotton Biology, State Key Laboratory of Crop Stress Adaptation and Improvement, Key Laboratory of Plant Stress Biology, Laboratory of Plant Germplasm and Genetic Engineering, Henan University, Kaifeng, 475004 Henan China

**Keywords:** Secondary metabolism, Transcriptional regulatory elements, Plant morphogenesis

## Abstract

Sweet osmanthus (*Osmanthus fragrans* Lour.) is among the top ten most well-known flowers in China and is recognized as both an aromatic plant and ornamental flower. Here, manual sectioning, scanning electron microscopy, and transmission electron microscopy of sweet osmanthus petals revealed that large amounts of lipids are present inside the petal cells and on the cell surfaces. However, no secretory structures were observed. Instead, the petal cells protrude slightly outward, and the surfaces of the cells are adorned with highly regular brush-shaped hairs. The surfaces of the ‘Yingui’ petals possessed mostly curled and more numerous hairs, whereas the ‘Dangui’ petals possessed fewer brush-shaped and more sparsely arranged hairs. In addition, many granular substances were attached to the brush-shaped hairs, and the granules were denser on the hairs of the ‘Yingui’ petals compared to the hairs on the ‘Dangui’ petals. Furthermore, 35 aromatic components in the ‘Yingui’ petals and 30 aromatic components in the ‘Dangui’ petals were detected via GC-MS. The main aromatic component of the ‘Yingui’ petals was β-ionone, whereas that of the ‘Dangui’ petals was linalool and its oxides. Transcriptome sequencing and qRT-PCR indicated that the high β-ionone content in the ‘Yingui’ petals was due to the overexpression of *CCD1* and *CCD4* and that the high linalool content in the ‘Dangui’ petals was due to the overexpression of *MECS*, *HDR*, *IDI1*, and *LIS1*, which function upstream of the linalool synthetic pathway. In particular, the expression levels of *CCD4* and *LIS1* were upregulated by 5.5- and 5.1-fold in the ‘Yingui’ and ‘Dangui’ petals, respectively. One transcription factor (ERF61) was cloned and named, and the expression pattern of *ERF61* in sweet osmanthus petals was found to be generally consistent with that of *CCD4*. Tobacco transformation experiments, yeast one-hybrid experiments, and electrophoretic mobility shift assays indicated that ERF61 binds to the *CCD4* promoter and stimulates *CCD4* expression, thereby regulating the synthesis of β-ionone in sweet osmanthus petals.

## Introduction

Floral fragrance, color, shape, surface structure, and nectar glands can affect the interactions of higher plants with pollinators, and floral fragrance, in particular, can also function to deter herbivores and attract natural enemies^1,2^. Floral scents generally contain complex volatile components, such as terpenoids and terpenoid derivatives, aromatic components, fatty acid derivatives, and other low-molecular-weight compounds, and the production and release of volatile oils from plant tissues are often associated with specialized tissues and organs, such as glandular hairs, oil glands, or resin tubes^2–4^. In some plants, such as members of Asclepiadaceae, Aristolochiaceae, Araceae, Burmanniaceae, and Orchidaceae, floral scents have been reported to originate from unique “aromatic glands”^5–10^.

In plants with aromatic glands, different parts of the flower can differentiate into flap-, cilia-, or hair-shaped glands that function in the production and emission of aromatic substances^9,10^. After volatile oils are produced in the secretory cells of aromatic glands, the oils are usually immediately released and disperse within a short period of time^5^. However, such oils can also form intracellular droplets. The mechanisms underlying floral scent production likely vary among plants and remain poorly understood.

*O. fragrans* is valued for its beauty and fragrance. The fragrance of the flower contains as many as 30 potentially exploitable chemical substances^11–13^. The plant has been cultivated for more than 2500 years in China and is among the top ten most well-known flowers in China. The flowering period, floral color, and other characteristics are used to classify sweet osmanthus cultivars into four groups: Yingui, Jingui, Dangui, and Sijigui^14,15^. Flowers in the Yingui group are relatively light in color (i.e., pale to medium yellow), whereas those of the Dangui group are relatively deep in color (i.e., orange to orange-red), and this difference is mainly attributed to differences in the levels of carotenoids, especially β-carotene^16,17^. The protein encoded by *CCD1* in sweet osmanthus is capable of cleaving carotenoids into ionones (e.g., α-carotene to α-ionone and β-ionone and β-carotene to β-ionone)^18,19^, whereas the protein encoded by *CCD4* can cleave β-carotene into β-ionone^20^. Previous studies of sweet osmanthus have also reported that the WRKY1 transcription factor regulates the expression of *CCD4* and might therefore regulate the cleavage of carotenoids and the synthesis of β-ionone, a key aromatic component^21^. Our previous study also revealed that the *CCD4* promoter region of sweet osmanthus contains multiple ERF transcription factor-binding elements and might therefore be regulated by an ERF transcription factor.

AP2/ERF transcription factors are a large family of transcription factors in plants that are named for their AP2/ERF domain, which is composed of 60–70 amino acids. They are generally separated into three subfamilies (AP2, ERF, and RAV) on the basis of sequence similarity and number of AP2/ERF domains^22^. ERF subfamily members contain a single AP2/ERF domain and can be further divided into two large subfamilies: the ERF subfamily and the CBF/DREB subfamily^23^. The *Arabidopsis thaliana* AP2/ERF transcription factor RAV1 recognizes CAACA and CACCTG elements, thereby regulating the expression of corresponding genes^24^. *CARAV1*, a new RAV gene, was isolated from pepper leaves that were infected with *Xanthomonas campestris* pv. *vesicatoria*. As in *A. thaliana*, the CARAV1 protein is able to recognize CAACA and CACCTG elements^25^, thereby regulating the expression of corresponding genes. In tobacco, an ERF transcription factor located at the NIC-2 locus is required to induce the jasmonate-mediated activation of nicotine biosynthesis^26^, and in *Catharanthus roseus*^27^, the jasmonate-responsive genes *ORCA2* and *ORCA3* control the expression of strictosidine synthase, which is required for the biosynthesis of terpenoid indole alkaloids.

*O. fragrans* is famous for its unique fragrance. Researching the production and regulatory mechanisms of the crucial floral scent components in sweet osmanthus could enrich our foundational knowledge of the molecular biology of sweet osmanthus, provide a theoretical basis for the utilization of primary floral scent components in sweet osmanthus, and improve the breeding of sweet osmanthus cultivars.

## Materials and methods

### Materials

Freshly cut flowering branches of *O. fragrans* ‘Baijie’ (Yingui group) and ‘Chenghong Dangui’ (Dangui group) were incubated at a constant temperature (22 °C) and relative humidity (70%) under a 12 h:12 h light:dark cycle (80 μmol m^−2^ s^−1^)^21,28^. Flowers at the full flowering stage were collected for analysis.

### Manual section detection

Fresh sweet osmanthus petals were manually sectioned, stained using 1% Sudan Black, and then observed and photographed using an Olympus BX60 microscope (Olympus, Tokyo, Japan).

### Scanning electron microscopy

Fresh petals were separated into two parts. One part was fixed in 2% glutaraldehyde for 3–5 h, washed using 0.1 M phosphate buffer, dehydrated using an ethanol gradient (30%, 50%, 70%, and 100%, successively), transferred to isoamyl acetate, and then subjected to critical-point drying using carbon dioxide. After being coated with gold, the petals were observed and photographed using a PHILIPS XL30ESEM environmental scanning electron microscope (ESEM) (Philips, Eindhoven, Netherlands). Meanwhile, the second part was directly adhered to a sample platform, subjected to ultralow-temperature freezing and coating with gold, transferred to a cold stage, and observed and photographed using a HITACHI S-3400N cryo-scanning electron microscope (cryo-SEM) (Hitachi, Tokyo, Japan).

### Transmission electron microscopy

Fresh sweet osmanthus petals were cut into 2 mm × 5 mm small blocks, fixed overnight in 1.25% paraformaldehyde and 1.25% glutaraldehyde (prepared using 0.1 M phosphate buffer, pH 7.0), washed using 0.1 M phosphate buffer, fixed in 1% osmic acid for 2.5 h, washed again using 0.1 M phosphate buffer, dehydrated using an ethanol gradient (30%, 50%, 70%, and 100%, successively), and then embedded in Epon812 epoxy resin. The embedded petals were then sectioned using a Leica ULTRACUT R ultramicrotome (Leica, Heidelberg, Germany), stained using uranyl acetate and lead citrate for 5–6 min, and observed and photographed using a JEM 100CX-II transmission electron microscope (TEM) (Jeol, Tokyo, Japan).

### Gas chromatography-mass spectrometer (GC-MS) analysis of sweet osmanthus petals

Fifty ‘Yingui’ and 50 ‘Dangui’ petals were separately sealed in 15 mL extraction flasks. After a 20-min equilibration period, a solid phase microextraction head was inserted, and after 40 min of headspace adsorption at 50–55 °C, the extraction head was removed, and the sample was loaded into the inlet port of the GC-MS system.

The chromatography conditions were as follows: TR-5MS (30 m × 0.25 mm × 0.25 μm) elastic quartz capillary column; a column flow of 1.1 mL/min; a temperature program of 50 °C for 2 min, an increase of 5 °C/min until reaching 120 °C, 120 °C for 2 min, an increase of 7 °C/min until reaching 220 °C, and 220 °C for 10 min; the carrier gas was highly pure helium (99.999%); and the splitless sample loading method.

The mass spectrometry conditions were as follows: Ei ion source; an ion trap temperature of 150 °C; a manifold temperature of 40 °C, a GC-MS transmission temperature (*I*/*F*) of 250 °C; a mass scanning range (*m*/*z*) of 35–600 u; an ionization energy of 70 eV; an emission current of 150 µA; and a detector voltage of 350 V. The experimental conditions were strictly controlled, and parallel experiments were performed in triplicate under identical conditions to analyze the accuracy and reliability of the solid phase microextraction (SPME) GC/MS data.

### RNA sequencing

RNA sequencing (RNA-Seq) was performed by BioMarker Technology (Beijing, China). The mRNA was isolated from petals, cut into shorter fragments, and ligated with adaptors. The first cDNA strand was produced using random primers and reverse transcription kits. The second cDNA strand was synthesized using DNA polymerase I, and adapter oligonucleotides with a hairpin loop structure were ligated to prepare them for hybridization. cDNA fragments of 150–250 bp were preferentially selected for sequencing on the Illumina HiSeq 2500 (Illumina, San Diego, USA) sequencing platform.

The expression levels of unigenes were calculated using the fragments per kilobase million (FPKM) method. Gene expression levels of differentially expressed genes (DEGs) were computed by the following formula: FPKM = cDNA fragments/[mapped fragments (millions) × transcript Length (kb)]. The FPKM results can be directly used to compare gene expression levels within differential samples.

### RNA extraction and cDNA cloning of the *ERF61* gene

Total RNA was extracted from flower petals using a MiniBEST universal RNA extraction kit (TaKaRa, Dalian, China) according to the manufacturer’s instructions. RNA quality and integrity were assessed using formamide denaturing gel electrophoresis, and RNA quantity was measured using spectrophotometry^29^. cDNA was then synthesized using a Superscript preamplification kit (Invitrogen, California, USA) according to the manufacturer’s instructions.

Forward (ERF61f1) and reverse (ERF61r1) primers were designed and used to amplify the full-length *ERF61* cDNA sequence under the following PCR amplification conditions: 94 °C for 4 min followed by 33 cycles of 94 °C for 30 s, 55 °C for 45 s, and 72 °C for 60 s. The PCR product was cloned into the pMD19-T vector (TaKaRa) and sequenced. Next, the *O. fragrans* ERF61 (OfERF61) amino acid sequence was compared to ERF sequences from other plant species. More specifically, the sequences were aligned using Clustal W version 1.83, phylogenetic analysis was performed using MEGA 4.1, and a neighbor-joining (NJ) tree was constructed using the distance matrix computed by MEGA.

### Reverse transcriptase-PCR, quantitative real-time PCR analysis

RNA was extracted from the petals of the ‘Dangui’ and ‘Yingui’ cultivars at the xiangyan stage (S1), initial flowering stage (S2), full flowering stage (S3), and late flowering stage (S4). Reverse transcriptase PCR (RT-PCR) and quantitative real-time PCR (qRT-PCR) primers are shown in Supplementary Table 1. The expression level of *ACTIN* was used as a reference, and RT-PCR amplification was performed using the following conditions: 94 °C for 4 min and 34 cycles of 94 °C for 30 s, 57 °C for 30 s, and 72 °C for 45 s. The reactions were performed in triplicate. qRT-PCR was performed using a Roche LightCycler 480II detection system, and each 20 µL reaction contained 10 µL SYBR Premix Ex Taq mix (Takara). Relative expression levels were calculated using the 2^−ΔΔCt^ method, and each analysis included three to five replicates.

### Transient transformation in tobacco leaves

A CCD4_pro_:GUS vector was constructed by using PCR and specific primers (CCD4f and CCD4r) to amplify a 976-bp *OfCCD4* promoter region (accession no. KF701121) and clone it into the pCAMBIA1391 vector. Meanwhile, the *OfERF61* cDNA sequence was amplified via PCR using gene-specific primers (ERF61f2 and ERF61f2) and cloned into the pHBT vector to construct the 35S_pro_:ERF61 vector. More specifically, the CCD4_pro_:GUS and 35S_pro_:ERF61 vectors were separately transformed into *A. tumefaciens* strain EHA105 using the freeze–thaw method, and EHA105 strains that contained the CCD4_pro_:GUS and 35S:ERF61 vectors were mixed at a 1:1 volume ratio, incubated at 25 °C for 2–4 h, and then cotransformed by transferring the vectors via a syringe onto the abaxial surface of fully expanded tobacco leaves. Leaves coinfiltrated with *Agrobacteria* carrying 35S:GFP and CCD4_Pro_:GUS vectors were used as controls. GUS activity was assayed by incubating tissue samples overnight at 37 °C in a 0.1% (w/v) X-Gluc solution that contained 50 mM sodium phosphate buffer (pH 7.2), 0.1% (w/v) Triton X-100, 2 mM K_3_Fe(CN)_6_, 2 mM K_4_[Fe(CN)_6_]·3H_2_O, and 10 mM EDTA. After staining, green tissues were bleached by soaking them for a few hours in 50% ethanol and were then washed using 70% ethanol.

### Yeast one-hybrid assay

The promoter region of the *OfCCD4* gene contains several CAACA elements (Supplementary Fig. 1), which are the binding sites of ERF transcription factors. Four copies of the CAACA element from the *OfCCD4* promoter were artificially synthesized into double-stranded DNA (C1), and both ends of the strand were modified with *Eco*RI and *Xho*I restriction endonuclease sites. The C1 strand was then annealed to *EcoR*I- and *Xho*I-digested pLacZi vectors to form the pLacZi-C1 recombinant vector. The *OfERF61* cDNA sequence was amplified using a *Xho*I site-containing forward primer (ERF61f3) and a *Bam*HI site-containing reverse primer (ERF61r3), purified, digested using *Bam*HI and *Xho*I, and then annealed to a *Xho*I- and *Bam*HI-digested pJG vector to form the pJG-ERF61 recombinant vector. Next, the pLacZi-C1 and pJG vector combination and pLacZi-C1 and pJG-ERF61 vector combination were cotransformed into YM4271 yeast cells. The resulting yeast cells were cultured in SD-Trp-Ura double-deficiency medium for 2–3 days, transferred to a nylon membrane, subjected to 2–3 freeze–thaw cycles in liquid nitrogen, and then incubated in staining buffer/X-gal solution for 30 min to 8 h at 30 °C.

### In vitro ERF61 protein synthesis and electrophoretic mobility shift assay

The *ERF61* cDNA sequence was amplified using an *Eco*RI site-containing forward primer (ERF61f4) and an *Xho*I site-containing reverse primer (ERF61r4), ligated into an *EcoR*I- and *Xho*I-digested pET-30a-c vector, and transformed into competent *Escherichia coli* BL21(DE3) cells. Protein expression and purification were then performed as described by Han et al.^21^, and the purified ERF61 protein was used for electrophoretic mobility shift assay (EMSA). More specifically, 3′ biotin-labeled sense oligonucleotides that contained both the CAACA element of the *OfCCD4* promoter and the corresponding antisense strand sequence were synthesized; the two oligonucleotides were annealed to form double-stranded DNA, and EMSA was performed using the LightShift Chemiluminescent EMSA Kit (Thermo Fisher, Waltham, USA) and Chemiluminescent Nucleic Acid Detection Module (Thermo Fisher, Waltham, USA), according to the manufacturer’s instructions.

## Results

### Structure of sweet osmanthus petals

**Fig. 1:**
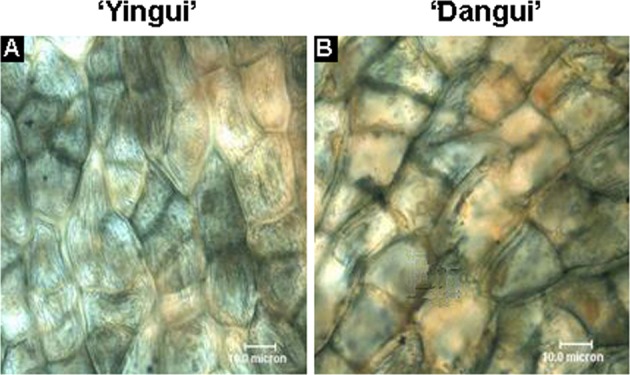
Manual section results of flower petals. **a** ‘Yingui’ and **b** ‘Dangui’

Sudan Blackdye is an efficient dye for lipid stains and dyes lipid granules in the cell a grayish-black, brown-black, or black color^30–33^. In this study, after Sudan Black staining, the petal cells appeared brown-black, and brown-black granules could be observed inside the cells (Fig. 1), which indicated that the petal cells contained large amounts of lipids.

**Fig. 2:**
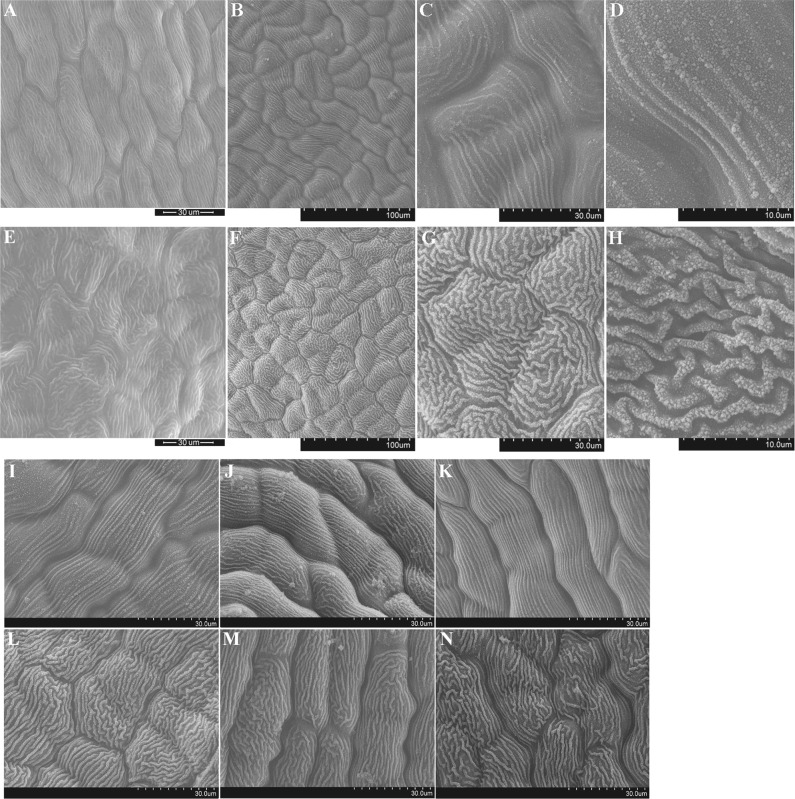
Results of scanning electron microscopy. **a**–**d** are full flowering stage petals of ‘Dangui’; **e**–**h** are full flowering stage petals of ‘Yingui’. **a** and **e** are results of ESEM; **b**–**d**, **f**–**h** are results of cryo-SEM; **i**–**k** are full flowering stage petals of three Dangui group cultivars. **i** ‘Zhushagui’, **j** ‘Yingye Dangui’, **k** ‘Zuijihong’; **l**–**n** are full flowering stage petals of three Yingui group cultivars. **l** ‘Zi Yingui’, **m** ‘Zaohuang’, **n** ‘Wan Yingui’

ESEM and cryo-SEM revealed an absence of secretory structures on the surface of the sweet osmanthus petals. Instead, the petal cells protruded slightly outward, and the cell surfaces of both cultivars were adorned with extremely regular brush-like strips known as brush-shaped hairs (Fig. 2a–h). However, the ‘Yingui’ petal surfaces possessed more densely arranged brush-shaped hairs that protruded significantly and were mostly curled (Fig. 2e–h), whereas the ‘Dangui’ petal surfaces possessed more sparsely arranged brush-shaped hairs that did not protrude and were nearly parallel in arrangement (Fig. 2a–d). Observation of the petal epidermis at high magnification also revealed that large numbers of granules were attached to the brush-shaped hairs, with some granules located between strips, and the granules on the ‘Yingui’ cultivar hairs were far greater in density and quantity than those of the ‘Dangui’ cultivar (Fig. 2c, d, g, h). Furthermore, the cryo-SEM results for the other three Dangui group cultivars (‘Zhushagui’, ‘Yingye Dangui’, and ‘Zuijihong’) (Fig. 2i–k) and the three Yingui group cultivars (‘Zi Yingui’, ‘Zaohuang’, and ‘Wan Yingui’) (Fig. 2l–n) were consistent with those of the two aforementioned cultivars. The differences in the epidermal cell morphology, density of brush-shaped hairs, and quantity of granules could be related to the different floral scent components and contents of the flower petals in the two cultivars.

**Fig. 3:**
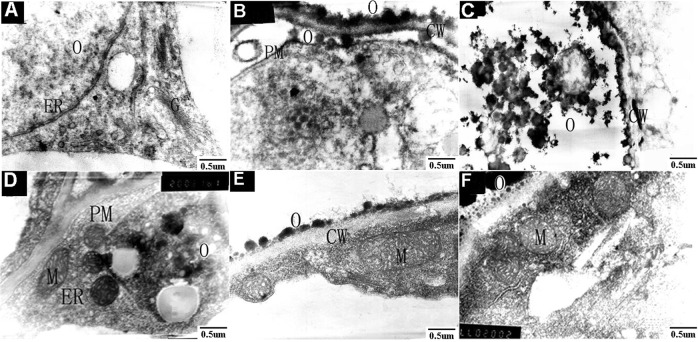
Results of transmission electron microscopy. **a**–**c** are ground petal tissues of ‘Yingui’ in the full flowering stage; **d**–**f** are ground petal tissues of ‘Dangui’ in the full flowering stage. ER endoplasmic reticulum, G Golgi apparatus, M mitochondrion, CW cell wall, O osmiophilic globules, PM plasma membrane

TEM confirmed that the sweet osmanthus petals lacked secretory structures and revealed that most of the petal cells contained electron-dense osmiophilic granules (Fig. 3a–f). Electron microscopy analysis revealed that the osmiophilic granule density on the surface and interior of the petal cells was higher in ‘Yingui’ compared to the ‘Dangui’ cultivar (Fig. 3b, e).

### Volatile aroma compounds of sweet osmanthus petals

GC-MS analysis revealed that 30 and 35 floral scent components were detected in the ‘Dangui’ and ‘Yingui’ petals, respectively, and two components, β-ionone and linalool, exhibited greater variation between the cultivars compared to the other scent components (Supplementary Fig. 2, Supplementary File 1). The components α-ionone, β-ionone, and dihydro-β-ionone accounted for 0.98%, 0.61%, and 0.38% of the total floral scent components, respectively, in the ‘Dangui’ cultivar but 7.7%, 35.59%, and 5.97%, respectively, in the ‘Yingui’ cultivar, with β-ionone exhibiting the greatest difference between the cultivars. In contrast, both *trans*-linalool (19.58%) and *cis*-linalool (25.26%) were more abundant in the ‘Dangui’ petals than in the ‘Yingui’ petals (2.31% and 2.77%, respectively).

### Linalool and ionone metabolism-related gene expression in sweet osmanthus petals

**Fig. 4:**
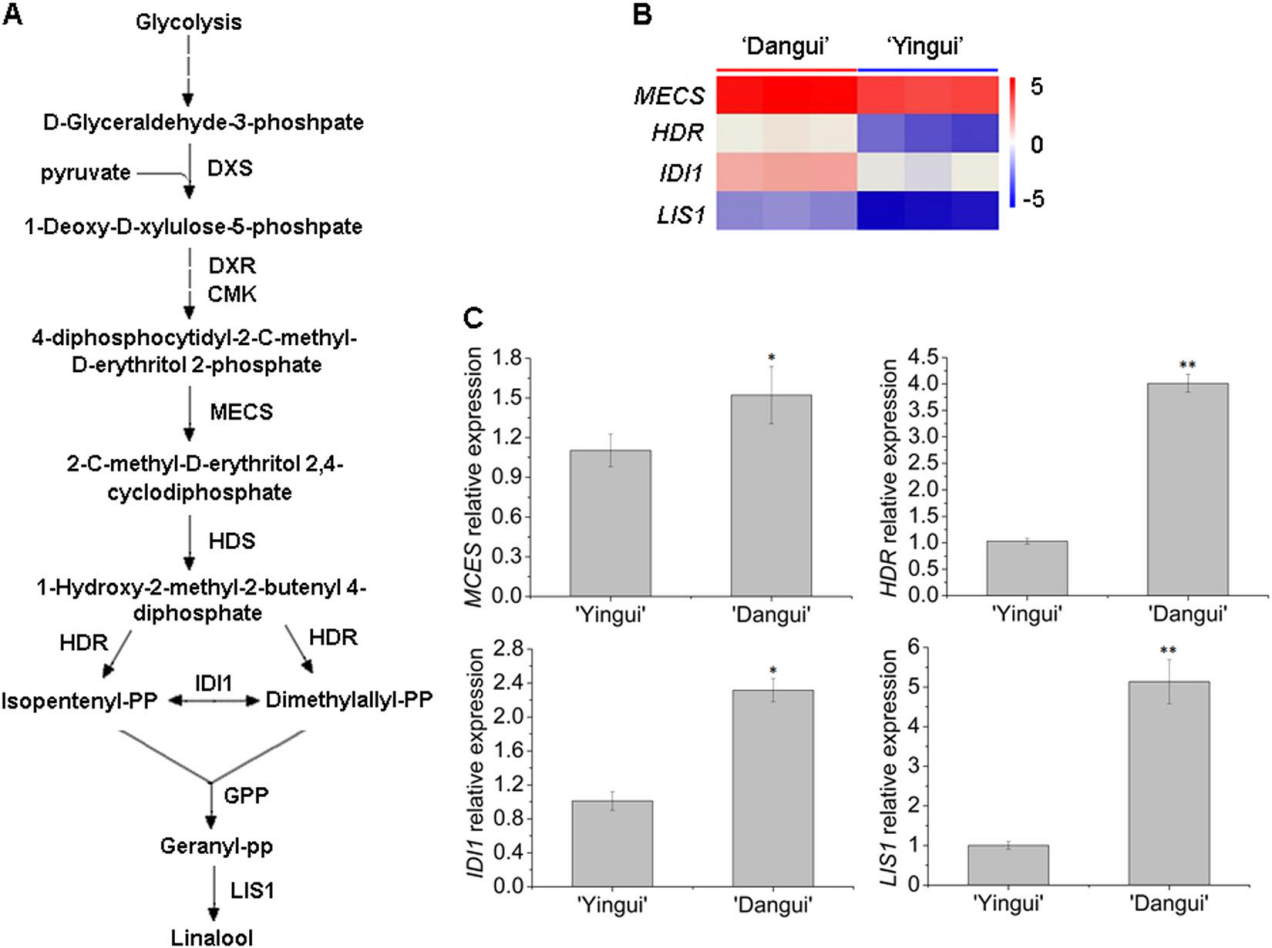
Transcript levels of selected genes involved in the linalool biosynthesis pathway in the full flowering stage petals of ‘Yingui’ and ‘Dangui’. **a** Linalool biosynthesis pathway. DXS 1-deoxy-d-xylulose 5-phosphate synthase, DXR 1-deoxy-d-xylulose 5-phosphate reductoisomerase, MCT 2-C-methyl-d-erythritol 4-phosphate cytidylyltransferase, CMK 4-(cytidine 5-diphospho)-2-C-methyl-d-erythritol kinase, MECS 2-C-methyl-d-erythritol 2,4-cyclodiphosphate synthase, HDS 4-hydroxy-3-methylbut-2-en-1-yl diphosphate synthase, HDR 4-hydroxy-3-methylbut-2-enyl diphosphate reductase, IDI isopentenyl pyrophosphate isomerase, GPP geranyl pyrophosphate synthase, LIS linalool synthase. **b** *MECS*, *HDR*, *IDI1,* and *LIS1* expression profiles from RNA-Seq. **c** qRT-PCR results of the *MECS*, *HDR*, *IDI1*, and *LIS1* genes. The qRT-PCR data represent the means ± SD of the three replicates from three independent experiments. *P *<* 0.05, **P *<* 0.01

Transcriptome sequencing revealed that four genes involved in the linalool synthetic pathway (*MECS*, *HDR*, *IDI1*, and *LIS1*) were significantly upregulated in the ‘Dangui’ petals compared to the ‘Yingui’ petals (Fig. 4b, Supplementary File 2), and qRT-PCR indicated that the expression levels of *MECS*, *HDR*, *IDI1*, and *LIS1* were 1.5-, 4.1-, 2.2-, and 5.1-fold greater in the ‘Dangui’ petals than in the ‘Yingui’ petals (Fig. 4c).

**Fig. 5:**
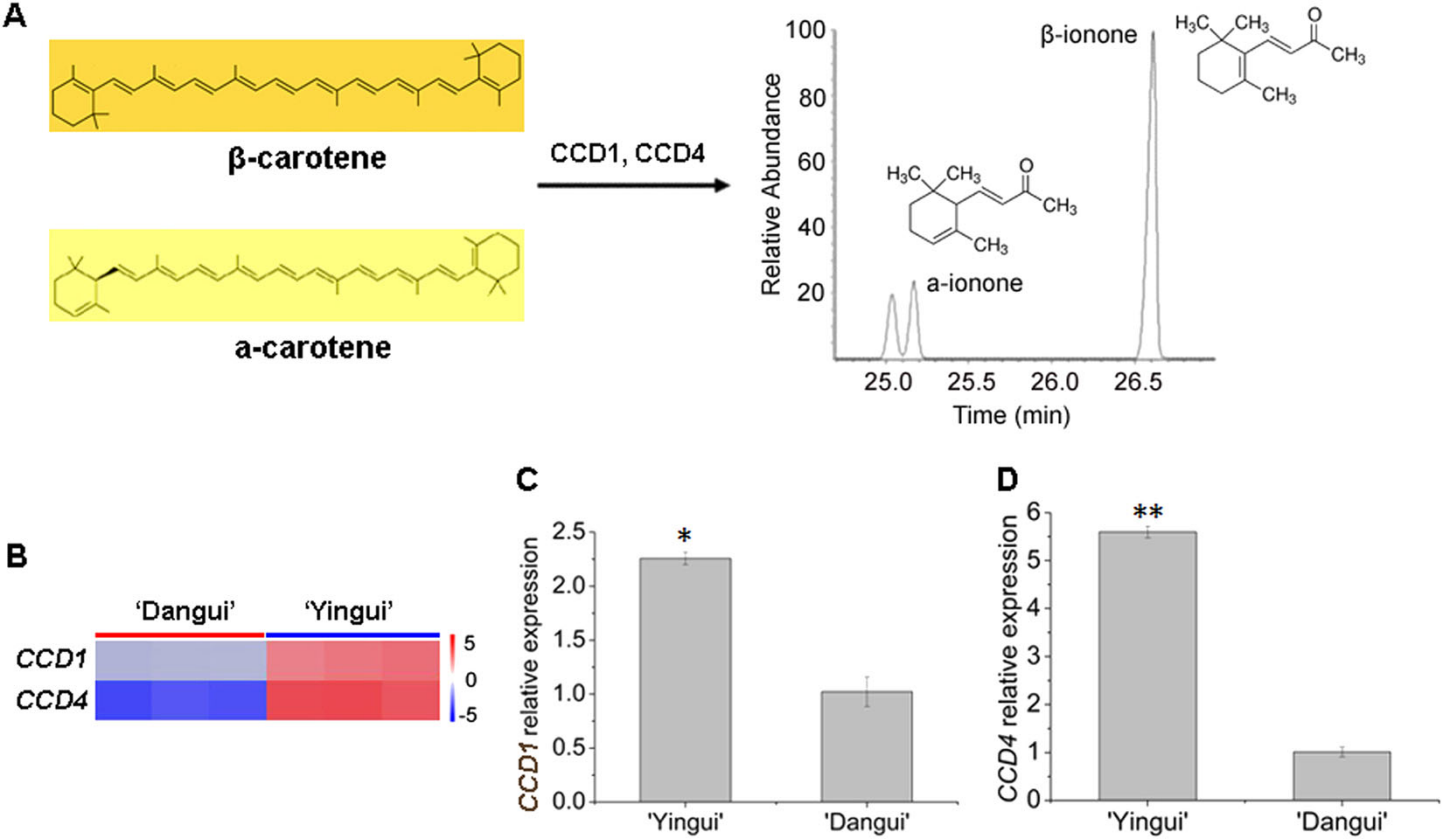
Transcript levels of the *CCD1* and *CCD4* genes in the petals of ‘Yingui’ and ‘Dangui’ in the full flowering stage. **a** CCD1 and CCD4 can cleave α-carotene and β-carotene into ionones; **b** *CCD1* and *CCD4* expression profiles from RNA-Seq; **c** qRT-PCR results for the *CCD1* and *CCD4* genes. The qRT-PCR data represent the means ± SD of the three replicates from three independent experiments. *P < 0.05, **P *<* 0.01

Both CCD1 and CCD4 are crucial for the cleavage of carotene into α-ionone and β-ionone (Fig. 5a). Transcriptome sequencing indicated that the expression levels of *CCD1* and *CCD4* were greater in the ‘Yingui’ petals than in the ‘Dangui’ petals (Fig. 5b, Supplementary File 2), and qRT-PCR indicated that the expression of the *CCD1* and *CCD4* genes was upregulated in the ‘Yingui’ petals 2.3- and 5.5-fold, respectively, compared to the expression of these genes in the ‘Dangui’ petals (Fig. 5c).

### Sequence analysis of *OfERF61* cDNA

The cDNA of *OfERF61* (GenBank accession no. MK994511) appeared to be a full-length sequence of 909 bp encoding a protein of 303 amino acid residues. Sequence alignment revealed that ERF61 shared high amino acid sequence identity with *Olea europaea* var. *sylvestris* ERF61, *Solanum lycopersicum* ERF61, and *Glycine max* ERF61 (Supplementary Fig. 3). A phylogenetic tree was constructed based on the amino acids of OfERF61 and other known ERFs from *A. thaliana*, and OfERF61 showed high similarity with AtERF55, AtERF59, and AtERF62 (Supplementary Fig. 4).

### Expression patterns of *ERF61* and *CCD4*

**Fig. 6:**
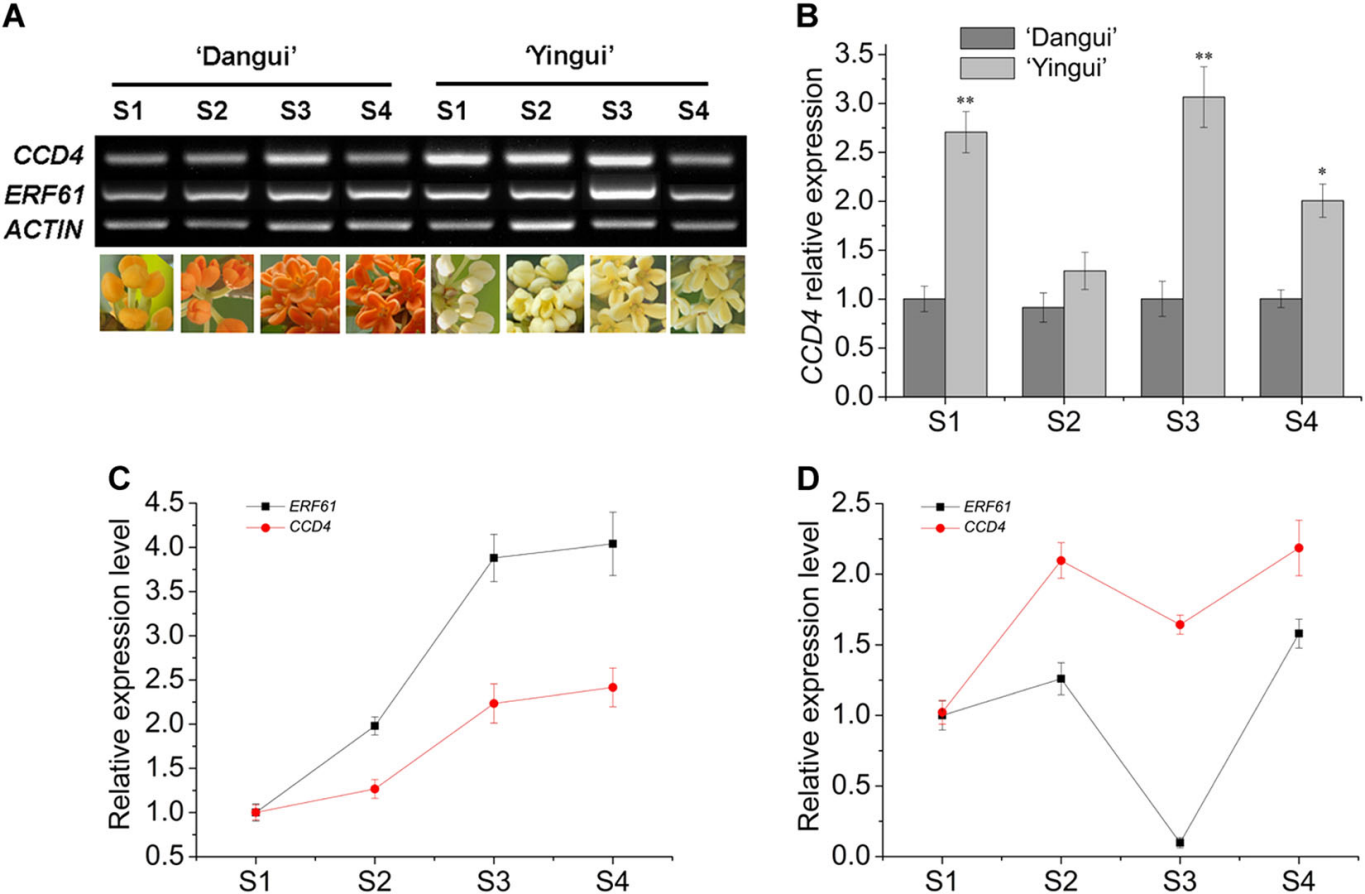
RT-PCR and qRT-PCR analysis of *ERF61* and *CCD4* transcripts in different flowering stages of ‘Dangui’ and ‘Yingui’. *ACTIN* transcripts were amplified as an internal control. **a** RT-PCR detection of *ERF61* and *CCD4* transcripts in different flowering stages of ‘Dangui’ and ‘Yingui’; **b** qRT-PCR analysis of *CCD4* transcripts in different flowering stages of ‘Dangui’ and ‘Yingui’; **c** qRT-PCR analysis of *ERF61* and *CCD4* transcripts in S1–S4 of ‘Yingui’; **d** qRT-PCR analysis of *ERF61* and *CCD4* transcripts in S1–S4 of ‘Dangui’. The qRT-PCR data represent the means ± SD of the three replicates from three independent experiments. *P < 0.05, **P *<* 0.01

RT-PCR and qRT-PCR were used to analyze samples from the two cultivars at different flowering stages. During the S1, S3, and S4 stages, the expression of *CCD4* in the ‘Dangui’ petals was significantly lower compared to the expression of *CCD4* in the ‘Yingui’ cultivar (Fig. 6a, b). However, the expression levels of *ERF61* and *CCD4* increased significantly from S1 to S4 in the ‘Yingui’ petals (Fig. 6c). In the ‘Dangui’ flower petals, the transcription levels of *ERF61* and *CCD4* increased significantly from S1 to S2, then gradually declined in S3, and returned to reach a peak at S4 (Fig. 6d). Although the *CCD4* and *ERF61* expression levels differed slightly in the two cultivars, the expression pattern of *ERF61* closely resembled that of *OfCCD4* (Fig. 6a, c, d). This suggests that *ERF61* may participate in *CCD4* regulation.

### Overexpression of *OfERF61* upregulates the transcript levels of *NbCCD4* in tobacco leaves

**Fig. 7:**
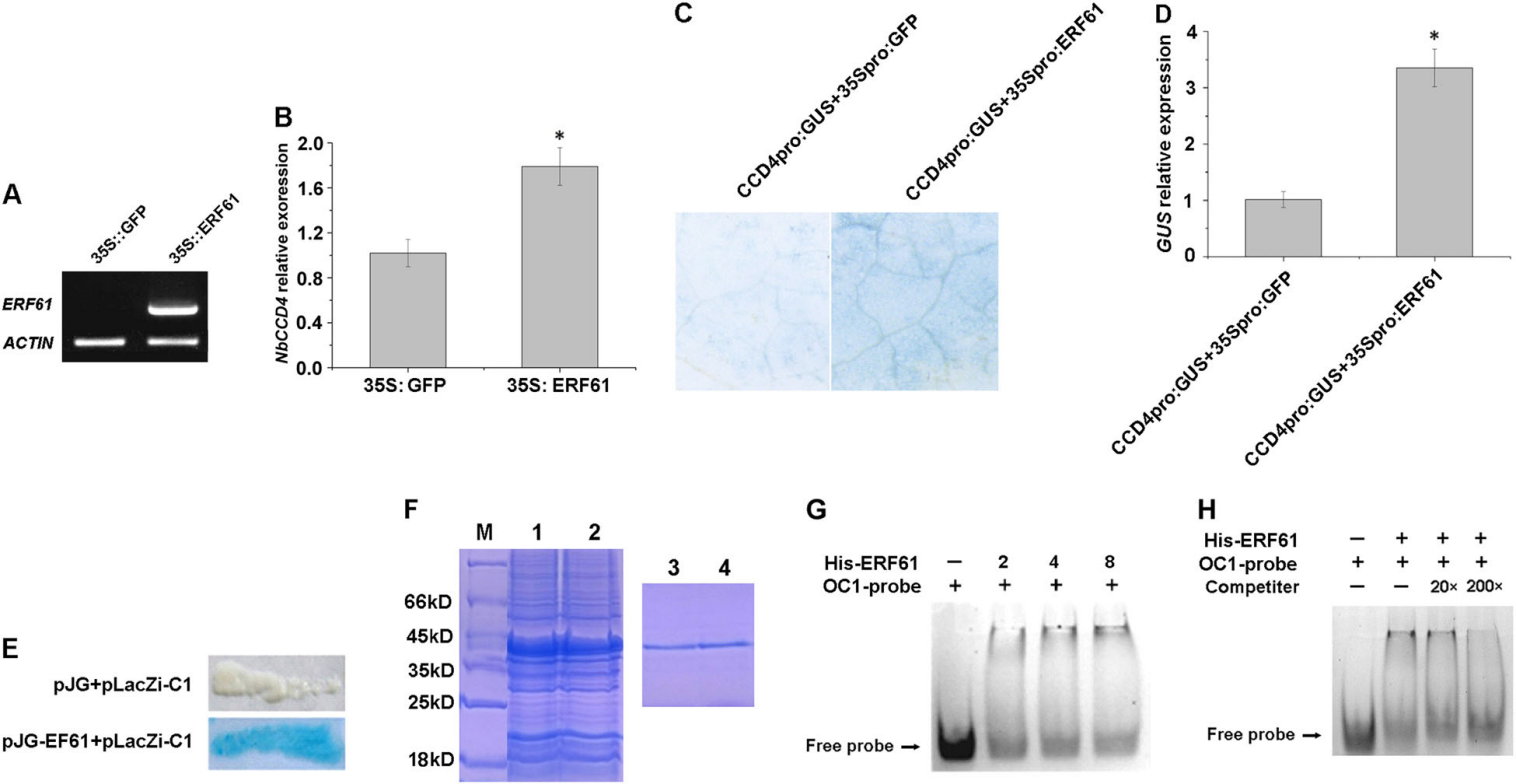
ERF61 can upregulate the transcript level of the *CCD4* gene by binding to and activating the *CCD4* promoter. **a** RT-PCR detection of the *ERF61* transcripts in tobacco leaves after coinfiltration with *Agrobacteria* carrying 35S:GFP and 35S:ERF61. **b** qRT-PCR analysis of the *NbCCD4* transcript levels in the control (35S:GFP) and transiently transformed tobacco leaves (35S:ERF61). **c** *CCD4* promoter activity in tobacco leaves after coinfiltration with *Agrobacteria* carrying the 35S:ERF61 and CCD4_Pro_:GUS vectors. Leave coinfiltration with *Agrobacteria* carrying 35S:GFP and CCD4_Pro_:GUS vectors was used as a control. **d** qRT-PCR analysis of *GUS* transcript levels in tobacco leaves after coinfiltration with *Agrobacteria* carrying 35S:ERF61 and CCD4_Pro_:GUS. Leave coinfiltration with *Agrobacteria* carrying 35S:GFP and CCD4_Pro_:GUS was used as a control. **e** β-Galactosidase activation was detected in yeast cotransformed with the pJG-ERF61 and pLacZi-C1 vectors. Yeast cotransformed with pJG and pLacZi-C1 was used as a control. **f** SDS-PAGE of proteins extracted from the cultured control *E. coli* cells transformed with *ERF61*-containing vectors (1 and 2 are two replicates) and SDS-PAGE of purified ERF61 recombinant protein by affinity chromatography (3 and 4 are two replicates). **g**, **h** Binding activity of ERF61 to the OC1-box element. **g** 0, 2,4, and 8 µg of ERF61 protein was added. **h** Competitors were added in 20- and 200-fold molar excess

To determine the ability of OfERF61 to upregulate transcript levels of the *CCD4* gene, *OfERF61* was transiently overexpressed in *Nicotiana benthamiana* leaves. Analyses of 35S:OfERF61-containing *A. tumefaciens*-infiltrated flowers relative to control 35S:GFP-infiltrated flowers revealed an increase in *OfERF61* transcription levels in the former (Fig. 7a). The transcription of *NbCCD4* was affected by the overexpression of *OfERF61*, with a 1.8-fold increase in *OfERF61-*overexpressing petals compared with the control (Fig. 7b).

### Interaction of ERF61 with the *CCD4* promoter

Promoter analysis of *CCD4* revealed that the region upstream of the *CCD4* promoter contained several CAACA-binding elements (Supplementary Fig. 1), suggesting that *CCD4* is regulated by the AP2/ERF transcription factor. When the combination of 35S_pro_:GFP and CCD4_pro_:GUS carrier (control) and the combination of 35S_pro_:ERF61 and CCD4_pro_:GUS carrier were transformed into the tobacco leaves (Fig. 7a), the cultured and stained leaves appeared blue. However, the color of the leaves in the control group was lighter, whereas the color of the tobacco leaves overexpressing *ERF61* was significantly deeper (Fig. 7c). qRT-PCR analysis of the tobacco leaves also indicated that *GUS* was significantly upregulated (3.37 times) in leaves that were cotransformed with 35S_pro_:ERF61 and CCD4_pro_:GUS carrier compared with the control group (Fig. 7d). In the yeast one-hybrid assay, the yeast cells that were cotransformed with pJG-ERF61 and the pLacZi-C1 carrier appeared blue after 20 min of staining, whereas the yeast cells in the control group, which were cotransformed with pJG and pLacZi-C1, did not appear blue, even after 8 h of staining (Fig. 7e). This suggests that *ERF61* binds to the *CCD4* promoter and thereby activates corresponding elements that increase *GUS* expression. The ERF61 protein was expressed in prokaryotes and purified (Fig. 7f). During EMSA, only one band was observed when the ERF61 protein was absent, whereas an obvious second band was observed when the OC1-labeled probe and ERF61 protein were added simultaneously. The additional band was the result of binding between the probe and the protein, and the amount of probe–protein complex gradually increased with increasing protein concentration (Fig. 7g). To verify the binding specificity of OfERF61, competition experiments were conducted by adding competitors (unlabeled OC1 probe) in 20- and 200-fold molar excess to the binding assay. The binding signals were reduced with the addition of 20-fold competitive unlabeled specific probe and not detected by adding 200-fold competitive unlabeled probe (Fig. 7h). These results suggest that ERF61 acts as a transcription factor and directly binds to the CAACA element that is upstream of the *CCD4* promoter, thereby positively regulating *CCD4* expression.

## Discussion

Previous studies have shown that the flower petals of sweet osmanthus contain many aromatic compounds, also called volatile oils^11–13^. The lipid granules in the plant cell can be dyed a grayish-black, brown-black, or black color using Sudan Black staining^30^. The present study used Sudan Black to stain sweet osmanthus petals. Both the epidermal and palisade cells of the petals were stained brown-black, and brown-black granules were also observed within the cells. This indicated that the sweet osmanthus petal cells contained large amounts of lipids, which could be related to the floral scent.

The synthesis of osmiophilic substances may involve the participation of Golgi bodies, the endoplasmic reticulum, plastids, and mitochondria. For example, the Golgi bodies and endoplasmic reticulum have been reported to participate in the synthesis of osmiophilic granules in the ciliated cells of *Typhonium venosum* (Araceae)^9^, and the present study revealed large numbers of osmiophilic granules in the petal cells of sweet osmanthus. The osmiophilic granules could be aggregates of aromatic substances. It is possible that such osmiophilic granules are transported outside the cell after synthesis and that the large numbers of osmiophilic granules observed on the cell walls may later be transported through cell gaps to the petal surface. This observation was confirmed by cryo-SEM, which revealed large numbers of granules on epidermal cells, especially on brush-shaped hairs. However, further research is needed to clarify the mechanism by which osmiophilic granules are synthesized and transported to the petal surface.

ESEM and cryo-SEM revealed strips of brush-shaped hairs on the surfaces of the petals of the ‘Dangui’ and ‘Yingui’ cultivars. This result was also confirmed by cryo-SEM analysis for the other Dangui and Yingui group cultivars. However, ESEM indicated that the brush-shaped hairs were smooth and that granules were absent, whereas cryo-SEM indicated that large numbers of granules were present on the brush-shaped hairs. This difference may have resulted from the sample preparation for ESEM, since granules on the petal surfaces might have fallen off after vacuum drying. The granules are aggregates of aromatic substances secreted by the sweet osmanthus petals and are very volatile. The number of granules on the ‘Yingui’ petal surfaces was far higher than that on the ‘Dangui’ petal surfaces, possibly because more aromatic components were produced by the ‘Yingui’ petals or because the aromatic components in the ‘Yingui’ petals were more prone to aggregation. The GC-MS results indicated that 35 and 30 scent components were present in the ‘Yingui’ and ‘Dangui’ petals, respectively. Furthermore, the ‘Yingui’ petals contained more β-ionone, whereas the ‘Dangui’ petals contained more linalool compounds. Since the molecular weight, density, flash point, and boiling point of linalool are lower than those of α-ionone and β-ionone (Table 1), linalool compounds tend to be more volatile, potentially resulting in fewer granules on the surface of ‘Dangui’ petals. However, the ‘Yingui’ petals contain more β-ionone, which is less volatile and tends to form granules.

**Table 1:** Chemical and physical properties of β-ionone, α-ionone, and linalool

	β-Ionone	α-Ionone	Linalool
CAS-No	14901-07-6	6901-97-9	78-70-6
Exact mass	192.15	192.15	154.25
Boiling point (°C)	262.95	259.5	198.5
Melting point (°C)	64.55	47.79	25
Flash point (°C)	122	122	78

The data from the SciFinder database

Most of the cells in sweet osmanthus petals are involved in the synthesis and secretion of aromatic substances and because they exhibit the characteristics of aromatic glands, these cells should be described accordingly. The entire sweet osmanthus petal can be considered a large aromatic gland since its basic tissues secrete aromatic substances that are then excreted by brush-shaped hairs on the cell surface.

Linalool synthase (LIS) uses geranyl pyrophosphate as a substrate and catalyzes the formation of linalool, a monoterpene alcohol with a sweet fragrance that occurs in floral scents of a wide variety of plants^34–36^. Constitutively high linalool emission has been engineered in several transgenic plant species by overexpressing the gene for *LIS*^37–40^, which is a key enzyme in the synthesis of linalool and its oxidized product. In the present study, transcriptome sequencing revealed that *LIS1* expression was significantly greater in the ‘Dangui’ petals than in the ‘Yingui’ petals (Fig. 4b), and qRT-PCR confirmed that the expression of *LIS1* in the ‘Dangui’ petals was 5.1 times greater compared to that in the ‘Yingui’ petals (Fig. 4c). Furthermore, transcriptome sequencing revealed that *MECS*, *HDR*, and *IDI1*, all of which function upstream of monoterpenoid metabolism, were upregulated in the ‘Dangui’ petals (Fig. 4b), and qRT-PCR confirmed that the three genes were upregulated by 1.5-, 4.1-, and 2.2-fold, respectively (Fig. 4c). These results indicate that the upregulation of *MECS*, *HDR*, *IDI1*, and especially *LIS1* ultimately increases the levels of linalools and oxidized linalools in the ‘Dangui’ petals to levels that are much higher than those present in the ‘Yingui’ petals.

Sweet osmanthus CCD1 can cleave α-carotene into α-ionone and β-ionone, whereas CCD4 can cleave β-carotene into β-ionone^18–20,41^. Both CCD1 and CCD4 are crucial for the cleavage of carotene into ionones. In the present study, the expression of both *CCD1* and *CCD4* in the ‘Yingui’ petals was significantly higher than that in the ‘Dangui’ petals, which indicated that the ‘Yingui’ petals should produce more α-ionone and β-ionone, as confirmed by GC-MS analysis. CCD4 mainly cleaves β-carotene into β-ionone. In addition, the expression of *CCD4* in the ‘Yingui’ petals was significantly higher compared to that in ‘Dangui’, regardless of the flowering stage. Therefore, *CCD4* is a crucial contributor to the greater β-ionone content in the ‘Yingui’ petals.

Previous studies have suggested that ERF transcription factors function either directly or in cooperation with other proteins or bind to relevant gene promoters, thereby regulating terpenoid metabolism in plants^42,43^. In *Artemisia annua*, AaEFR1 and AaEFR2 have been reported to bind to CBF2 and RAV1AAT elements on the promoters of *ADS* and *CYP71AV1*, respectively, thereby regulating the synthesis of artemisinin^44^. In *C. roseus*, the AP2/ERF transcription factors OCA3 and OCA4 bind to the promoters of *TDC*, *STR*, *CPR*, and other genes involved in the terpenoid indole alkaloid metabolic pathway^45^. In the present study, the expression of the cloned ERF transcription factor gene *ERF61* was generally identical to that of *CCD4* during the flowering stages, regardless of cultivar. The transcription of *N. benthamiana CCD4* was affected by overexpression of *OfERF61*, with a 1.8-fold increase in *OfERF61-*overexpressing tobacco leaves compared to the control. Therefore, the ERF61 transcription factor may regulate the expression of *CCD4*. The tobacco transformation experiments revealed that ERF61 binds to the *CCD4* promoter, and the yeast one-hybrid and EMSA experiments revealed that ERF61 binds to the CAACA element in the *CCD4* promoter. Together, these results suggest that ERF61 regulates the expression of *CCD4*, thereby regulating the cleavage of carotenoids and synthesis of β-ionone in sweet osmanthus petals and ultimately regulating the floral scent of sweet osmanthus. In this study, we confirmed that *OfCCD4* was regulated by ERF61. The *CCD4* gene may be regulated by other activator proteins. Future studies should endeavor to identify the regulating factors involved in the cleavage of carotenoids and further to elucidate the β-ionone biosynthesis machinery.

### Data Archiving Statement

We have read and understood your journal’s policies, and we believe that neither the manuscript nor the study violates any of these policies.

## Supplementary information

Phylogenetic relationships of ERF61 with some *A. thaliana* ERF proteins

Primers used in this study

Comparison of aroma components in ‘Dangui’ and ‘Yingui’

Information for the identified linalool and ionone metabolism-related upregulated unigenes

The promoter region of *CCD4* gene

GC-MS fingerprints of ‘Yingui’ and ‘Dangui’

Sequence alignment and phylogenetic analysis of ERF61 with some ERF61 proteins

Supplementary Information
