# Supplementary figures and images for: Mechanism of floral scent production in Osmanthus fragrans and the production and regulation of its key floral constituents, β-ionone and linalool
Source: Hortic Res. 2019 Sep 7;6:106. doi: 10.1038/s41438-019-0189-4 (PMC6804851; doi:10.1038/s41438-019-0189-4)

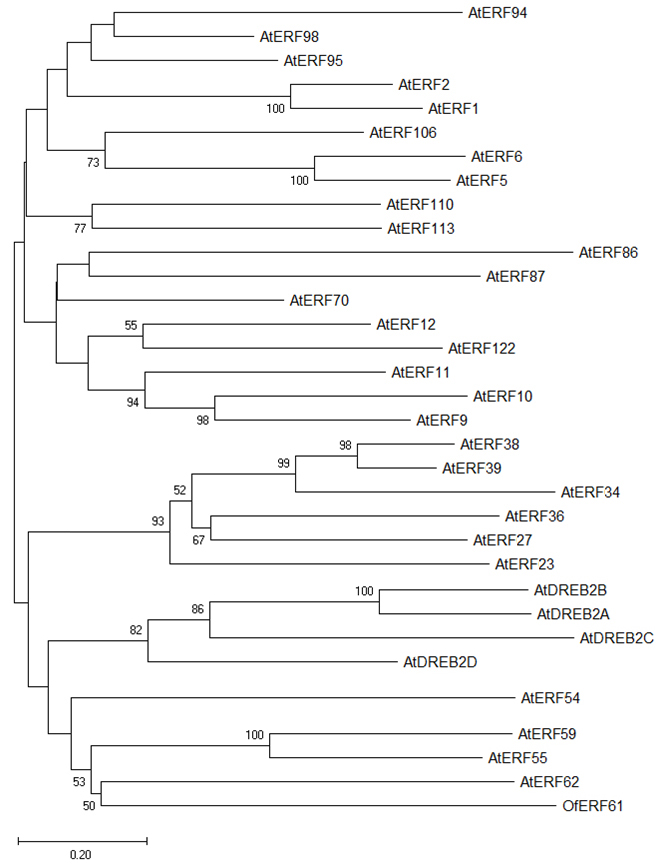

Supplement: Supplementary file 1 — Phylogenetic relationships of ERF61 with some A. thaliana ERF proteins [file 41438_2019_189_MOESM1_ESM.jpg]

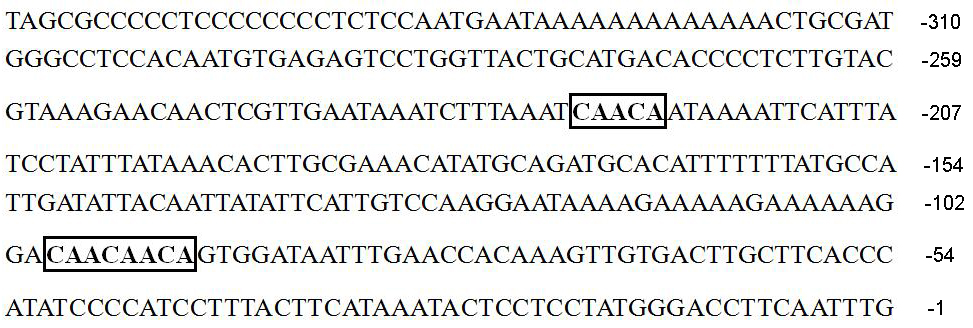

Supplement: Supplementary file 5 — The promoter region of CCD4 gene [file 41438_2019_189_MOESM5_ESM.jpg]

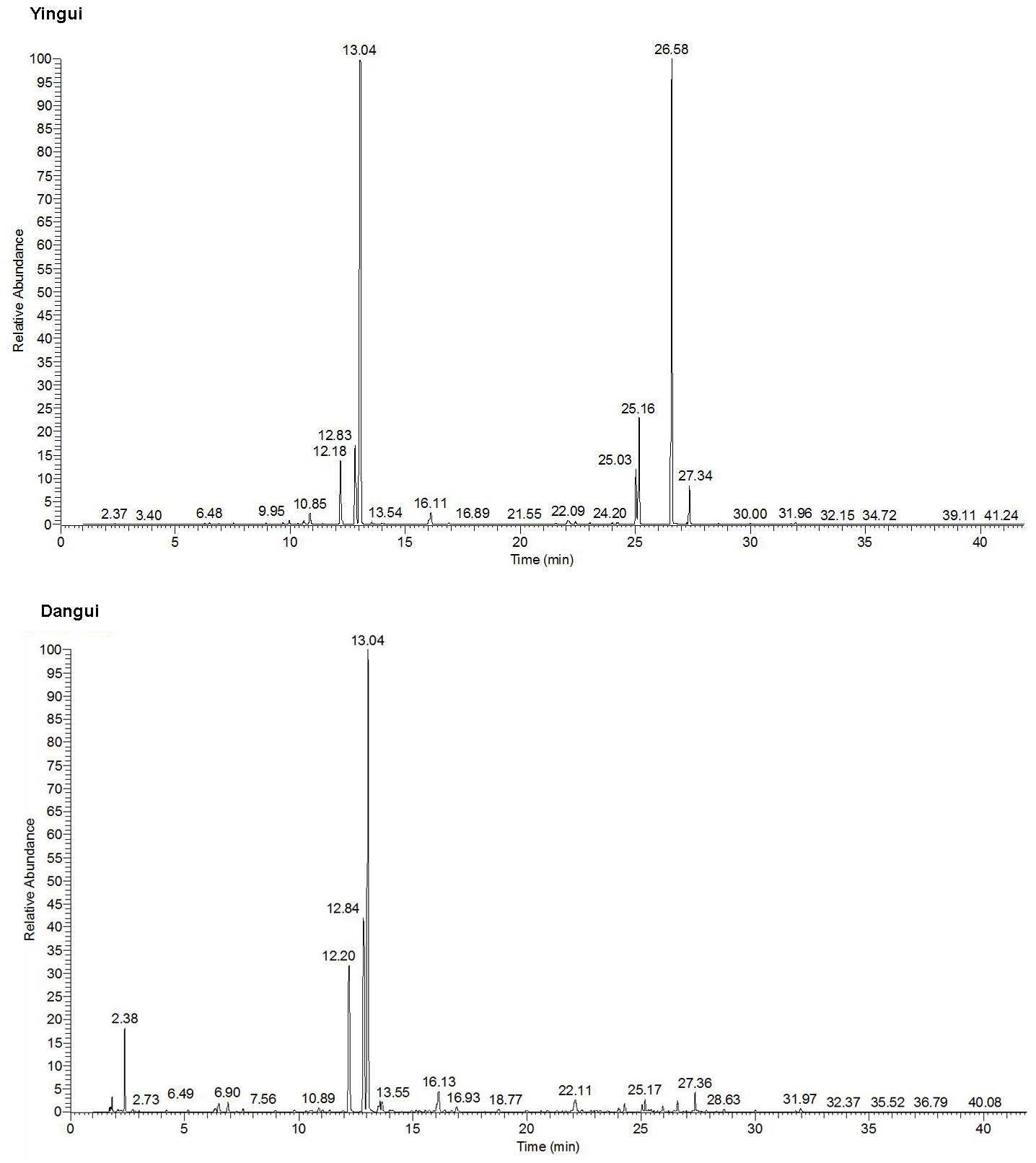

Supplement: Supplementary file 6 — GC-MS fingerprints of ‘Yingui’ and ‘Dangui’ [file 41438_2019_189_MOESM6_ESM.jpg]

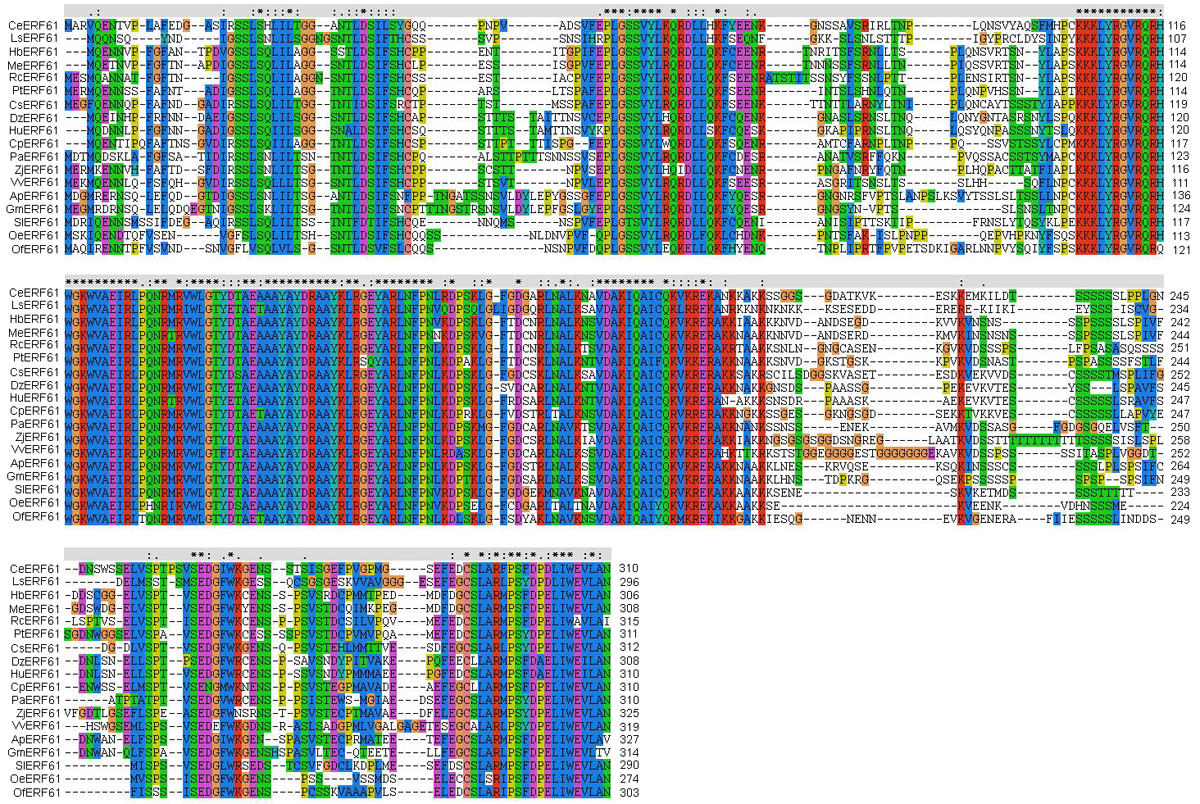

Supplement: Supplementary file 7 — Sequence alignment and phylogenetic analysis of ERF61 with some ERF61 proteins [file 41438_2019_189_MOESM7_ESM.jpg]
