# Supplementary material for: Mechanism of floral scent production in Osmanthus fragrans and the production and regulation of its key floral constituents, β-ionone and linalool
Source: Hortic Res. 2019 Sep 7;6:106. doi: 10.1038/s41438-019-0189-4 (PMC6804851; doi:10.1038/s41438-019-0189-4)
Supplement: Supplementary file 2 — Primers used in this study [file 41438_2019_189_MOESM2_ESM.docx]

| **Supplementary Table 1 Primers used in this study** | |
| --- | --- |
| **Primer name** | **Primers for amplification (5’-3’)** |
| ERF61f1 | ATGGCCCAAATTCGAGAAAACAAC |
| ERF61r1 | ATTAGCAAGAACTTCCCATATCA |
| ERF61-RT-f | CCATTACGAAAACCAAACAAACC |
| ERF61-RT-r | CATCAACAGAGTTCTTCACAGCG |
| CCD4-RT-f | CACCTCAGCCAGTAAAGAAAC |
| CCD4-RT-r | TGAAAACACATTAGGAACAAC |
| Ofactin-f | CGTGGCACTTGACTATGAA |
| Ofactin-r | TCTGGGCAACGGAATCTCT |
| Nbactin-f | ATCCTCACAGAGCGTGGTTAC |
| Nbactin-r | CACTGAGCACTATGTTTCCGT |
| CCD4f | GCGAAGCTTCGTGCTATCAAACGGAGTCTTAT |
| CCD4r | GCGGGATCCTGTTTCTCTTCTTGTTAGCTTGC |
| ERF61f2 | GCGAAGCTTATGGCCCAAATTCGAGAAAACAA |
| ERF61r2 | GGCACTAGTATTAGCAAGAACTTCCCATATCA |
| ERF61f3 | CGGCTCGAGATGGCCCAAATTCGAGAAAACAA |
| ERF61r3 | GGCGGATCCATTAGCAAGAACTTCCCATATCAA |
| ERF61f4 | GCGGAATTCATGGCCCAAATTCGAGAAAACAA |
| ERF61r4 | GGCCTCGAGATTAGCAAGAACTTCCCATATCAA |
| C1-s | AATTTCAACAATCAACAATCAACAATCAACAA |
| C1-r | TCGATTGTTGATTGTTGATTGTTGATTGTTGA |
| OC1-labeled probe-s | AAAGAAAAAAGGACAACAACAGTGGATAATTTGA |
| OC1-labeled probe-r | TCAAATTATCCACTGTTGTTGTCCTTTTTTCTTT |
| GUS-f1(qRT-PCR) | GGGTGAAGGTTATCTCTATGAA |
| GUS-r1(qRT-PCR) | GTAAAGTAGAACGGTTTGTGGT |
| CCD4-f1(qRT-PCR) | CATTTTCTCTCCAATCCCCTT |
| CCD4-r1(qRT-PCR) | TGGTGGTTTGTGGTTTATCTT |
| CCD1-f1(qRT-PCR) | CTTGTCGCCTTGAGAATCCAG |
| CCD1-r1(qRT-PCR) | AGTGTAACTTTCGTTCACCCT |
| ERF61f5(qRT-PCR) | AGGAGTGAGGCAGCGACAATG |
| ERF61r5(qRT-PCR) | CGTATTCGCCACGGAGTTTAT |
| Ofactin-f1(qRT-PCR) | GTGGCACTTGACTATGAA |
| Ofactin-r1(qRT-PCR) | TCTGGGCAACGGAATCTCT |
| Nbactin-f1(qRT-PCR) | CCACGAGACCACATACAACTCT |
| Nbactin-r1(qRT-PCR) | CTATCGGCAATACCTGGGAACA |
| NbCCD4f(qRT-PCR) | TACCACCAAACAAACAGTAGAGC |
| NbCCD4r(qRT-PCR) | TCAATGAAAGCGTTCACGAAA |
| MECS-f(qRT-PCR) | ACTTGGATGCCACTTTGATTT |
| MECS-r(qRT-PCR) | AGGTTTACGGCTGAAGGGTCT |
| HDRf(qRT-PCR) | GGTGTTTCGGCACAACTTGAC |
| HDRr(qRT-PCR) | GTACTCGTACCCATTCTCCTT |
| IDI1f(qRT-PCR) | CACGAACTTGATTACCTGCTC |
| IDI1r(qRT-PCR) | GCTTAATACCACCCTCACCAG |
| LISf(qRT-PCR) | TGACTGATCCCAGCTTGTCGG |
| LISr(qRT-PCR) | GGGTGAGTTCATCGGGTTTCC |
