# Supplementary material for: Mechanism of floral scent production in Osmanthus fragrans and the production and regulation of its key floral constituents, β-ionone and linalool
Source: Hortic Res. 2019 Sep 7;6:106. doi: 10.1038/s41438-019-0189-4 (PMC6804851; doi:10.1038/s41438-019-0189-4)
Supplement: Supplementary file 8 — Supplementary Information [file 41438_2019_189_MOESM8_ESM.docx]

Supplementary Table 1 Primers used in this study

Supplementary File 1 Comparison of aroma components in 'Dangui' and 'Yingui'

Supplementary File 2 Information for the identified linalool and ionone metabolism-related upregulated unigenes

Supplementary Fig. 1 The promoter region of the *CCD4* gene. 'CAACA' elements are marked in a box.

Supplementary Fig. 2 GC-MS fingerprints of the aroma components in 'Dangui' and 'Yingui' flower petals.

Supplementary Fig. 3 Sequence alignment and phylogenetic analysis of ERF61 with other ERF61 proteins. The deduced amino acid sequence of OfERF61 is aligned with homologs from *Citrus sinensis* (CsERF61; accession no. XP_006476394.1), *Populus trichocarpa* (PtERF61; accession no. XP_002304554.1), *Hevea brasiliensis* (HbERF61; accession no.XP_021642650.1), *Ricinus communis* (RcERF61; accession no.XP_002509951.1), *Coffea eugenioides* (CeERF61; accession no.XP_027182874.1), *Durio zibethinus* (DzERF61; accession no.XP_022738829.1), *Olea europaea* var. *sylvestris* (OeERF61; accession no.XP_022886965.1), *Manihot esculenta* (MeERF61; accession no.XP_021604440.1), *Carica papaya* (CpERF61; accession no.XP_021894819.1), *Lactuca sativa* (LsERF61; accession no.XP_023746139.1), *Prunus avium* (PaERF61; accession no.XP_021818645.1), *Solanum lycopersicum*(SlERF61; accession no.XP_004245520.1), *Ziziphus jujuba* (ZjERF61; accession no.XP_015896421.1), *Abrus precatorius* (ApERF61; accession no.XP_027361214.1), *Glycine max* (GmERF61; accession no.XP_003516719.1), *Vitis vinifera* (VvERF61; accession no.RVX04153.1), *Herrania umbratica* (HuERF61; accession no.XP_021280351.1).

Supplementary Fig. 4 Phylogenetic relationships of ERF61 with *A. thaliana* ERF proteins. The accession nos. of AtERF62, AtERF55, AtERF59, AtERF54, AtERF1, AtERF2, AtERF94, AtERF95, AtERF98, AtERF5, AtERF6, AtERF106, AtERF110, AtERF113, AtERF11, AtERF12, AtERF70, AtERF86, AtERF87, AtERF122, AtERF9, AtERF10, AtERF34, AtERF36, AtERF38, AtERF39, AtERF23, AtERF27, AtDREB2A, AtDREB2B, AtDREB2C, and AtDREB2D are Q9SVQ0, Q9SKW5, Q8H1E4, Q9M0J3, AEE76738, AED95487, Q9LND1, Q9LTC6, Q9LTC5, AED95489, AEE83902, Q9LY05, Q70II3, Q9LYU3, Q9C5I3, Q94ID6, Q9C995, Q6J9Q2, Q9FZ90, Q38Q40, Q9FE67, Q9ZWA2, Q8LBQ7, Q9LU18, Q9ZQP3, Q9SUK8, Q1ECI2, Q38Q39, AED90871, AEE74994, OAP10757 and Q9LQZ2, respectively. The analysis was performed by the NJ method using MEGA version 4.1. Bar, 0.2 substitutions per site.
